# Clinical Characteristics of Patients with Different N-Terminal Probrain Natriuretic Peptide Levels after Hematopoietic Stem Cell Transplantation

**DOI:** 10.1155/2020/8839336

**Published:** 2020-10-16

**Authors:** Zhen Se, Haobin Zhou, Hanlin Li, Jing Sun, Qiong Zhan, Qingchun Zeng, Qifa Liu, Dingli Xu

**Affiliations:** ^1^State Key Laboratory of Organ Failure Research, Department of Cardiology, Nanfang Hospital, Southern Medical University, Guangzhou, China; ^2^Key Laboratory for Organ Failure Research, Ministry of Education of the People's Republic of China, Guangzhou, China; ^3^Department of Hematology, Nanfang Hospital, Southern Medical University, Guangzhou, China

## Abstract

Heart failure (HF) is not uncommon among patients with hematologic malignancies (HM) undergoing hematopoietic stem cell transplantation (HSCT) and is associated with an increased mortality. Among HSCT patients without signs or symptoms of HF, groups with elevated and normal N-terminal probrain natriuretic peptide (NT-proBNP) levels have been poorly characterized in previous literature. Herein, we reviewed consecutive admissions for HM undergoing HSCT (*n* = 301). Based on NT-proBNP levels and clinical signs or symptoms of HF at follow-up (one month after HSCT), patients were grouped into ENPH (*e*levated *N*T‐proBNP > 125 pg/mL, *p*resence of *H*F symptoms or signs), ENAH (*e*levated *N*T‐proBNP > 125 pg/mL, *a*bsence of *H*F symptoms or signs), and NN (*n*ormal *N*T‐proBNP < 125 pg/mL). ENPH, ENAH, and NN were observed in 22.9%, 54.5%, and 22.6% of patients, respectively. ENPH patients had a significantly higher baseline NT-proBNP level, followed by the ENAH and NN groups, respectively (*P* < 0.001). Frequencies of HLA partially matched related donors, stem cell source (bone marrow+peripheral blood), and utilization of graft-versus-host disease prophylaxis regimens (ciclosporin+methotrexate+antithymocyte globulin±mycophenolate mofetil) were also the highest in the ENPH group, followed by ENAH and NN groups, respectively (all *P* < 0.05). Uric acid and hemoglobin levels, transplant type, and cyclophosphamide-based conditioning regimens utilized were similar between the ENAH and ENPH groups. We found that ENPH and ENAH are commonly observed in HM hospitalized for HSCT. Serum NT-proBNP levels may allow for earlier identification of HSCT patients at high risk of developing cardiac dysfunction.

## 1. Introduction

The increasing use of hematopoietic stem cell transplantation (HSCT) as a curative strategy for hematologic malignancies has favorably modified the prognosis of hematologic malignancies [1–5].

Heart failure (HF) is a serious complication of HSCT affecting approximately 5% to 43% of HSCT patients and is associated with increased mortality [6, 7]. Several clinical factors have been identified that may contribute to HF. These include pre-HSCT exposures, HSCT conditioning regimens, and graft-versus-host disease (GVHD) [8, 9]. Previous studies have characterized and identified powerful predictors of HF in patients with hematologic malignancies undergoing HSCT [10–15]. However, the diagnostic criteria for HF utilized thus far have been based on reduced left ventricular ejection fraction (LVEF) and clinical signs or symptoms. While a decline in LVEF may represent a late marker of cardiotoxicity [16], a normal LVEF does not exclude the presence of early HF or HF with preserved LVEF (HFpEF). Given that HFpEF constitutes between 22 and 73% of HF patients in the general population (HFpEF), a significant number of patients might have been excluded from analysis [17–19]. Recently, the importance of brain natriuretic peptide (BNP) or N-terminal probrain natriuretic peptide (NT-proBNP) as a biomarker for the diagnosis and prognostic evaluation of HF has been emphasized in European, U.S., and Chinese guidelines [17, 20, 21]. While some patients that present with elevated BNP or NT-proBNP levels may not meet the diagnostic criteria for HF (i.e., patients with no clinical symptoms or signs or with no apparent structural or functional cardiac abnormalities), they may still be at an increased risk for cardiotoxicity. Data on this group of patients is scarce.

In the current study, we aimed to describe and compare the clinical characteristics of HSCT patients with elevated NT-proBNP levels but without clinical symptoms or signs of HF, those with elevated NT-proBNP levels and symptoms or signs of HF, and patients with normal NT-proBNP levels after HSCT.

## 2. Methods

### 2.1. Study Population

**Figure 1:**
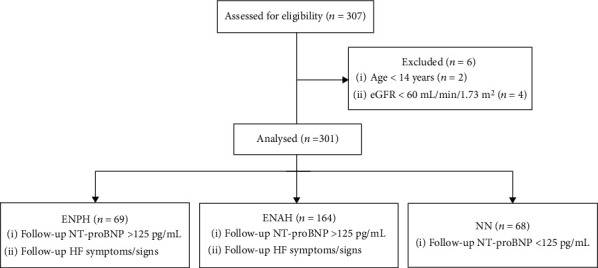
Patient flow chart. eGFR: estimated glomerular filtration rate; NT-proBNP: N-terminal probrain natriuretic peptide; HF: heart failure; ENPH: elevated NT‐proBNP > 125 pg/mL, presence of HF symptoms or signs; ENAH: elevated NT‐proBNP > 125 pg/mL, absence of HF symptoms or signs; NN: normal NT‐proBNP < 125 pg/mL.

This retrospective single-center cohort study included consecutively admitted patients who underwent autologous and allogeneic HSCT for hematologic malignancies between January 2015 and December 2016 at Nanfang Hospital, Southern Medical University. Inclusion into this study required complete baseline (within 24 h of admission) and follow-up (one month post-HSCT) pairs of NT-proBNP levels. Patients who were <14 years of age or have estimated glomerular filtration rate (eGFR) < 60 mL/min/1.73m^2^ were excluded (Figure 1). Major diagnoses included acute myeloid leukemia (AML) (*n* = 130), acute lymphoblastic leukemia (ALL) (*n* = 107), acute mixed lineage leukemia (*n* = 8), biphenotypic acute leukemia (*n* = 7), myelodysplastic syndrome (MDS) (*n* = 13), chronic myeloid leukemia (CML) (*n* = 7), and lymphoma (*n* = 16). Overall, 259 (86.0%) patients received allogeneic and 42 (14.0%) received autologous HSCT. The study was approved by the institutional review board of Nanfang Hospital. Written informed consent was obtained from all participants.

Demography, medical history, physical examination, laboratory tests, admission electrocardiogram, chest X-ray, echocardiography, and transplantation-related data (diagnosis, type of transplant, donor type, stem cell source, conditioning regimen, and prophylaxis regimens for GVHD) were collected retrospectively from the hospital clinical database for analysis. Twelve-lead electrocardiography and chest X-ray were performed in all patients at baseline. Echocardiographic data was available in 285 patients (95%). The assessment of LVEF by echocardiography was standardized. Recommendations for optimal therapy were based on guidelines. Patients received sodium phosphocreatine as part of their cardioprotective therapy.

### 2.2. Transplant Procedure

Conditioning regimens mainly consisted of busulfan (BU), cyclophosphamide (CY), total body irradiation (TBI), anthracycline (i.e., daunorubicin), etoposide (VP-16), fludarabine (FLU), and cytarabine among others. A combination of ciclosporin A (CsA), methotrexate (MTX), antithymocyte globulin (ATG), and mycophenolate mofetil (MMF) was given for prophylaxis against GVHD. Stem cell sources included bone marrow and peripheral blood stem cells.

### 2.3. Definitions

Body mass index (BMI, kg/m^2^) was calculated as the body weight (kg) divided by height (m) squared. eGFR was calculated using the Modification of Diet in Renal Disease equation [22]. Anemia was defined as hemoglobin < 13 g/dL for men and <12 g/dL for women, according to the World Health Organization criteria. Blood samples for measurements of NT-proBNP were taken within 24 h of admission (baseline) and at follow-up. The patient population was stratified into 3 groups based on their NT-proBNP levels and concomitant HF symptoms and/or signs at follow-up (Figure 1): patients with elevated NT-proBNP (NT‐proBNP > 125 pg/mL) and presence of HF symptoms or signs (ENPH); patients with elevated NT-proBNP (NT‐proBNP > 125 pg/mL) but absence of HF symptoms or signs (ENAH); and patients with normal NT-proBNP (NT‐proBNP < 125 pg/mL, NN). The cut-off value for NT-proBNP was chosen according to the 2016 ESC guidelines on HF [17].

### 2.4. Statistical Analysis

Normally distributed continuous variables were presented as mean ± SD, while nonnormal data was presented as median and interquartile range. Categorical variables were presented as percentages. Differences between groups were tested by the chi-square test for categorical data and analysis of variance or the Kruskal-Wallis test for continuous data, as appropriate. A two-tailed *P* value < 0.05 was considered statistically significant. Analyses were performed using SPSS version 20.0 (IBM SPSS Statistics, IBM Corporation, Armonk, New York).

## 3. Results

A total of 301 patients were included in the study. Of those, 69 patients (22.9%) had ENPH, 164 patients (54.5%) had ENAH, and 68 patients (22.6%) had NN. Characteristics of the patients are presented in Table 1. The mean age was 32.4 ± 11.7 years and 40.2% were females. The mean LVEF at admission for the overall population was 61.1 ± 9.3%. Median NT-proBNP levels increased from 55 (30.0, 112.0) pg/mL at baseline to 415 (141.0, 1283.0) pg/mL at one month for the overall population (*P* < 0.001). ENPH showed the highest baseline and post-HSCT NT-proBNP levels, followed by the ENAH and NN groups. ENAH resembled ENPH regarding uric acid and hemoglobin levels (*P* < 0.05). Baseline systolic and diastolic blood pressure, heart rate, LVEF, and eGFR were comparable between groups (*P* > 0.05).

Table 2 summarizes transplantation-related data. Patients with ENAH were more likely to suffer from ALL. Allogeneic transplantations, frequencies of HLA partially matched related donors, and stem cell source (bone marrow+peripheral blood) were the highest in the ENPH group, followed by ENAH and NN groups, respectively (all *P* < 0.05). ENAH resembled ENPH with regard to transplant type and treatment with CY-based conditioning regimen. The use of anthracycline did not differ among the three groups. Regarding the prophylaxis regimens for GVHD, the frequency of CsA+MTX+ATG±MMF administration in ENPH was the highest, followed by ENAH and NN (*P* < 0.05). CsA+MTX±MMF use was similar among the three groups (*P* > 0.05).

## 4. Discussion

This study compared the clinical characteristics between patient subgroups defined by an elevated NT-proBNP and presence of signs or symptoms of HF (ENPH), elevated NT-proBNP but absence of signs or symptoms of HF (ENAH), and normal NT-proBNP levels (NN). Herein, we observed that patients with ENPH and ENAH represented a substantial proportion of HSCT hospitalizations (22.9% and 54.5%, respectively). ENAH, a traditionally understudied subgroup, appeared to have many features that were intermediary in the transition between ENPH and NN, while simultaneously resembling HF with respect to many clinical characteristics.

Previous studies have described characteristics of patients with and without heart failure who underwent HSCT and identified powerful predictors of HF post-HSCT. However, these studies failed to differentiate between patients with ENAH and those with NN. Whether patients with ENAH have the same characteristics and experience the same mortality risks or benefits as those with ENPH and NN, respectively, has not been explored thus far. The three classification systems used herein were designed to cover a broader spectrum transitioning from ENPH, through a state of ENAF, to NN, to better represent the HSCT population and characterize the ENAH group.

Early detection of therapy-induced myocardial injury, although challenging, is crucial, as initiation of treatment may prevent progression of HF. Furthermore, those at higher risk for cardiotoxicity may benefit from preemptive cardioprotective interventions. However, there is a lack of established consensus on optimal strategies to detect cardiotoxicity. Traditionally, echocardiography has been used to assess cardiotoxicity [23]. However, because of the insensitivity of the LVEF response to cardiotoxicity, a large proportion of patients may have sustained irreversible myocardial injury when changes in LVEF become apparent, thus highlighting the need for biomarkers. Cardiac biomarkers, such as BNP/NT-proBNP or cardiac troponins, have proven to be a reliable diagnostic tool for the early detection, assessment, and monitoring of chemotherapy-induced cardiotoxicity [24]. A normal BNP level in an untreated patient virtually excludes significant cardiac disease, making even an echocardiogram unnecessary [25]. However, the role of these cardiac biomarkers in routine monitoring to define high-risk patients in the context of HSCT is not well established.

NT-proBNP is considered superior to other indicators in the early detection of myocardial injury based on its favorable sensitivity and specificity. Therefore, the present study used NT-proBNP levels to stratify patients and detect myocardial injury. This is consistent with 2016 ESC HF guidelines for the general population [17]. We found NT-proBNP levels increased significantly between admission and one month post-HSCT, suggesting therapy-induced myocardial injury is a common complication of HSCT.

ENAH, a precursor to overt HF, may represent mild myocardial injury or an early-stage HF. In the general population, asymptomatic left ventricular dysfunction is common and carries a poor prognosis [26]. In a community-based study enrolling 4257 participants, Wang et al. found that subjects with mild asymptomatic left ventricular systolic dysfunction (ALVD) had a 2- to 4-fold increase in the rate of chronic HF and mortality compared with subjects with normal systolic function [26]. Thus, identification of this precursor is important as initiation of treatment at this stage may decrease mortality in these high-risk patients [27]. A recent study reported a 5.1% incidence of ALVD in patients with lymphoma undergoing autologous HSCT [15]. However, this patient group was not characterized in detail. In this study, we observed a high prevalence of ENAH. Our results showed that higher baseline NT-proBNP levels with both the ENPH and ENAH groups resulted in higher NT-proBNP post-HSCT. This emphasizes that an asymptomatic rise in NT-proBNP (i.e., ENAH) may still increase the risk of cardiac dysfunction and supports that the ENAH group may benefit from further targeted intervention. Herein, a second assessment of echocardiographic function on one-month follow-up was not available. Therefore, we were unable to assess changes in left ventricular function of these patients. Speculatively, a repeated assessment of echocardiographic function in HSCT patients with NT‐proBNP > 125 pg/mL may provide further insights.

The prevalence of ENPH was 22.9% in the present study, which is consistent with values reported in previous studies [10, 11]. In this study, both groups with cardiac impairment after HSCT had higher baseline NT-proBNP and lower hemoglobin levels than the NN group, implying that greater baseline disease severity relates to subsequent cardiotoxicity. Furthermore, the ENPH group experienced a greater increase in NT-proBNP from baseline to post-HSCT compared with the ENAH group. This demonstrates that patients with HF signs or symptoms suffer from a greater exacerbation of cardiotoxicity than those with asymptomatically increased NT-proBNP.

Various HSCT-related exposures have been reported to contribute to cardiac dysfunction during HSCT [8, 9]. Anthracycline and CY are both known to exhibit dose-dependent cardiotoxicity [8, 28] and reportedly increase the risk of HF [13, 29, 30]. In our study, patients in the ENAH and ENPH groups were more frequently receiving CY, while anthracycline use was comparable between groups. The absence of exact doses of conditioning regimens limits the ability to determine relationships between anthracycline or CY doses and cardiac function.

It is important to note that cardiac dysfunction in hematological malignancies may be related to factors other than HSCT-related exposures such as tumor cell invasion, anemia, and electrolyte disorders. Additionally, a gender disparity in the prevalence of HF has also been observed in HSCT in previous research [13, 31, 32]. Consistently, we found the association between female gender and an increased incidence of ENPH was tending toward significance. The underlying mechanism of gender-specific cardiotoxicity is incompletely understood. It has been reported that differences in body composition between genders may affect the disposition and the subsequent metabolism of anthracyclines [32]. Also, a greater burden of diastolic HF in women may explain this observation.

## 5. Limitations

The study was a retrospective observational single-center study. Due to the observational nature of this study, it is impossible to prove causality. We did not have data on the dose of chemotherapeutic agents. Echocardiographic data was only obtained at admission and not at follow-up; however, other pertinent variables were available at admission and follow-up.

## 6. Conclusion

This study found that ENPH and ENAH are commonly observed in patients with hematologic malignancies hospitalized for HSCT. Some characteristics of ENAH resemble ENPH, while others represent an intermediate in the transition between ENPH and NN. Herein, by using NT-proBNP levels for stratification, the characteristics of patients across a broad spectrum of cardiovascular dysfunction are observed. This may allow earlier identification of patients at high risk of developing cardiac dysfunction and extends the window of opportunity for clinical interventions. Further studies are needed to better characterize these patients and identify those who are more likely to develop HF and could benefit from closer monitoring and early intervention.

## Figures and Tables

**Table 1:** Patient characteristics.

Variables	Total (*n* = 301)	ENPH (*n* = 69)	ENAH (*n* = 164)	NN (*n* = 68)	*P* value
Age (years)	32.4 ± 11.7	31.8 ± 11.5	32.5 ± 11.7	32.7 ± 12.1	0.893
Female	121 (40.2)	32 (46.4)	70 (42.7)	19 (27.9)	0.056
BMI (kg/m^2^)	21.1 ± 3.7	21.5 ± 3.3	20.8 ± 3.5	21.6 ± 4.4	0.237
Haemodynamics					
Systolic BP (mmHg)	114.5 ± 10.6	114.3 ± 10.5	114.7 ± 10.9	114.3 ± 10.0	0.958
Diastolic BP (mmHg)	70.5 ± 12.5	70.4 ± 12.3	70.6 ± 12.7	70.3 ± 12.6	0.985
Heart rate (beats/min)	83.5 ± 11.9	83.64 ± 13.3	83.8 ± 11.7	82.5 ± 11.0	0.740
LVEF (%)	61.1 ± 9.3	60.3 ± 8.7	61.10 ± 9.4	61.92 ± 9.6	0.633
LVEDD (mm)	43.0 ± 4.60	42.9 ± 3.9	42.87 ± 4.8	43.28 ± 4.9	0.827
Laboratory values					
Sodium (mEq/L)	137.5 ± 3.0	137.9 ± 3.3	137.6 ± 3.0	136.9 ± 2.7	0.149
Uric acid (mg/dL)	3.4 ± 1.6	3.3 ± 1.5	3.2 ± 1.5	3.86 ± 1.8	0.011
eGFR (mL/min/1.73m^2^)	161.2 ± 59.7	149.3 ± 48.9	163.7 ± 59.7	167.0 ± 68.2	0.159
NT-proBNP (pg/mL) (baseline)	55.0 (30.0, 112.0)	70.0 (31.0, 119.0)	59.0 (37.25, 119.0)	33.50 (23.8, 55.0)	<0.001
NT-proBNP (pg/mL) (post-HSCT)	415.0 (141.0, 1283.0)	1055.0 (374.0, 5155.0)	538.5 (254.5, 1366.0)	55.0 (38.0, 81.3)	<0.001
Hemoglobin (g/dL)	89.2 ± 20.7	83.4 ± 18.6	87.1 ± 19.9	100.0 ± 20.9	<0.001

Values are mean ± SD, *n* (%), or median (interquartile range). ENPH: elevated NT-proBNP and presence of clinical signs or symptoms of heart failure; ENAH: elevated NT-proBNP but absence of symptoms or signs of heart failure; NN: normal NT-proBNP; BMI: body mass index; BP: blood pressure; LVEF: left ventricle ejection fraction; LVEDD: left ventricular end-diastolic diameter; eGFR: estimated glomerular filtration rate; NT-proBNP: N-terminal probrain natriuretic peptide.

**Table 2:** In-hospital transplantation-related parameters.

Variables	Total (*n* = 301)	ENPH (*n* = 69)	ENAH (*n* = 164)	NN (*n* = 68)	*P* value
Diagnosis					
Acute lymphoblastic leukemia	107 (35.5)	16 (23.2)	70 (42.7)	21 (30.9)	0.012
Acute myeloid leukemia	130 (43.2)	36 (52.2)	62 (37.8)	32 (47.1)	0.099
Other^a^	64 (21.3)	17 (24.6)	32 (19.5)	15 (22.1)	0.672
Transplant type					0.033
Autologous HSCT	42 (14.0)	7 (10.1)	19 (11.6)	16 (23.5)	
Allogeneic HSCT	259 (86.0)	62 (89.9)	145 (88.4)	52 (76.5)	
Donor type					
HLA matched, related	119 (39.5)	22 (31.9)	63 (38.4)	34 (50.0)	0.087
HLA partially matched, related	99 (32.9)	32 (46.4)	56 (34.1)	11 (16.2)	0.001
HLA matched, unrelated	30 (10.0)	4 (5.8)	19 (11.6)	7 (10.3)	0.402
Stem cell source					<0.001
Peripheral blood	198 (65.8)	37 (53.6)	101 (61.6)	60 (88.2)	
Bone marrow+peripheral blood	103 (34.2)	32 (46.4)	63 (38.4)	8 (11.8)	
Conditioning regimens					
CY based	267 (88.7)	64 (92.8)	152 (92.7)	51 (75.0)	<0.001
Anthracycline based	51 (16.9)	14 (20.3)	28 (17.1)	9 (13.2)	0.545
TBI based	138 (45.8)	25 (36.2)	85 (51.8)	28 (41.2)	0.063
TBI+CY+VP-16/FA	113 (37.5)	23 (33.3)	69 (42.1)	21 (30.9)	0.198
Prophylaxis regimens for GVHD					
CsA+MTX±MMF	110 (36.8)	19 (27.9)	63 (38.4)	28 (41.8)	0.202
CsA+MTX+ATG±MMF	141 (47.2)	40 (58.8)	79 (48.2)	22 (32.8)	0.010

Values are mean ± SD, *n* (%). ENPH: elevated NT-proBNP and presence of clinical symptoms or signs of heart failure; ENAH: elevated NT-proBNP but absence of symptoms or signs of heart failure; NN: normal NT-proBNP; HSCT: hematopoietic stem cell transplantation; HLA: human leukocyte antigen; CY: cyclophosphamide; TBI: total body irradiation; BU: busulfan; VP-16: etoposide; FA: fludarabine and cytarabine; GVHD: graft-versus-host disease; CsA: ciclosporin A; MTX: methotrexate; MFF: mycophenolate mofetil; ATG: antithymocyte globulin. ^a^Lymphoma (*n* = 16), myelodysplastic syndrome (*n* = 13), acute mixed lineage leukemia (*n* = 8), chronic myeloid leukemia (*n* = 7), biphenotypic acute leukemia (*n* = 7), etc.

## Data Availability

The data used to support the findings of this study are available from the corresponding author upon request.
